# Succinate Dehydrogenase Deficiency in Pediatric and Adult Gastrointestinal Stromal Tumors

**DOI:** 10.3389/fonc.2013.00117

**Published:** 2013-05-17

**Authors:** Martin G. Belinsky, Lori Rink, Margaret von Mehren

**Affiliations:** ^1^Department of Medical Oncology, Fox Chase Cancer CenterPhiladelphia, PA, USA

**Keywords:** gastrointestinal stromal tumor, wild type, succinate dehydrogenase, insulin-like growth factor receptor, review

## Abstract

Gastrointestinal stromal tumors (GISTs) in adults are generally driven by somatic gain-of-function mutations in KIT or PDGFRA, and biological therapies targeted to these receptor tyrosine kinases comprise part of the treatment regimen for metastatic and inoperable GISTs. A minority (10–15%) of GISTs in adults, along with ∼85% of pediatric GISTs, lacks oncogenic mutations in KIT and PDGFRA. Not surprisingly these wild type (WT) GISTs respond poorly to kinase inhibitor therapy. A subset of WT GISTs shares a set of distinguishing clinical and pathological features, and a flurry of recent reports has convincingly demonstrated shared molecular characteristics. These GISTs have a distinct transcriptional profile including over-expression of the insulin-like growth factor-1 receptor, and exhibit deficiency in the succinate dehydrogenase (SDH) enzyme complex. The latter is often but not always linked to bi-allelic inactivation of *SDH* subunit genes, particularly *SDHA*. This review will summarize the molecular, pathological, and clinical connections that link this group of SDH-deficient neoplasms, and offer a view toward understanding the underlying biology of the disease and the therapeutic challenges implicit to this biology.

## Introduction

Gastrointestinal stromal tumors (GISTs) are the most common sarcomas of the digestive tract, with an estimated annual incidence of 11–19.6 cases per million (Corless et al., 2011). GISTs generally present as sporadic disease in older adults, with a median age of diagnosis of 63 and 59 years in two large series of GISTs presenting in the stomach and small intestine, respectively (Miettinen et al., 2005b, 2006b). In these reports from the Armed Forces Institute of Pathology, civilian-only GIST cases are relatively gender-balanced, with a male to female ratio of 48.7:51.3% (*n* = 2109). Though GISTs occur throughout the GI tract, by far the most common sites are in the stomach (60%) and intestine (30%) (Demetri et al., 2010). These predominantly spindle-cell tumors are thought to originate from the interstitial cells of Cajal (ICC), which promote the autonomous peristaltic contractions of the gut. GISTs and ICC share morphological and immunophenotypic characteristics, most notably the expression of the KIT receptor tyrosine kinase (RTK) (Kindblom et al., 1998; Sircar et al., 1999). At the molecular level, 85–90% of GISTs possess mutually exclusive gain-of-function mutations in KIT or in the related RTK PDGFRA (Hirota et al., 1998, 2003; Rubin et al., 2001; Heinrich et al., 2003), while ∼10–15% lack these kinase mutations and are known as wild type (WT) GIST. Secondary large-scale cytogenetic changes, such as loss of chromosome 14q, 22q, and 1p, occur frequently in the progression of mutant GIST (El-Rifai et al., 2000a; Debiec-Rychter et al., 2001; Assamaki et al., 2007; Gunawan et al., 2007; Wozniak et al., 2007). Mutations in *KIT* occur most frequently in exon 11, encoding the juxtamembrane domain of the receptor, or in exon 9, which encodes the extracellular dimerization domain, while mutations in the tyrosine kinase domain (exon 18) is the most common *PDGRA* event (Corless et al., 2011). Recently, mutations in the serine-threonine protein kinase BRAF have also been reported as rare events in GIST (Agaram et al., 2008b; Agaimy et al., 2009; Belinsky et al., 2009; Hostein et al., 2010; Corless et al., 2011). Treatment for advanced GIST has been greatly enhanced by the tyrosine kinase inhibitors (TKI) imatinib mesylate (IM; Gleevec, Novartis) and sunitinib (Sutent, Pfizer), which target the constitutively active mutant isoforms of KIT and PDGFRA.

Gastrointestinal stromal tumors are exceedingly rare in children and adolescents. In the two large series mentioned above, GIST patients under 21 years of age constituted only 2.7% of gastric and 0.6% of intestinal GIST cases (Miettinen et al., 2005b, 2006b). The United Kingdom National Registry of Childhood Tumors reported an annual incidence of 0.02 per million for children under the age of 14 (Benesch et al., 2009). Pediatric GISTs exhibit distinct clinical, pathological, and molecular characteristics as compared to adult tumors. GISTs in this age group occur more often in girls, generally present in the stomach as multi-focal nodular growth, and usually lack activating kinase mutations and cytogenetic aberrations seen in adult GISTs (Janeway et al., 2007; Agaram et al., 2008a; Belinsky et al., 2009; Benesch et al., 2009; Pappo and Janeway, 2009). Pediatric and adult WT GISTs express high levels of another RTK, the insulin-like growth factor-1 receptor (IGF1R) (Prakash et al., 2005; Agaram et al., 2008a; Tarn et al., 2008; Janeway et al., 2010), which may represent an alternate therapeutic target in this subset of GISTs.

A subset of adult WT GISTs shares the distinct clinico-pathological and molecular characteristics of pediatric tumors; these tumors are sometimes described as “Type II” or “pediatric-like” GISTs to delineate them from the majority of adult sporadic tumors (Gill et al., 2010b; Rege et al., 2011). Included under this umbrella classification are GISTs from multi-neoplastic syndromes known as Carney triad (CT) and Carney–Stratakis syndrome (CSS). The unifying molecular observation for this group of tumors was recently shown to be a deficiency in the mitochondrial succinate dehydrogenase (SDH) complex, often but not always attributable to inactivating mutations in *SDH* component genes (Gill et al., 2010b; Gaal et al., 2011; Janeway et al., 2011; Miettinen et al., 2011; Belinsky et al., 2012; Doyle et al., 2012). The mitochondrially located SDH complex oxidizes succinate to fumarate within the Krebs/tricarboxylic acid (TCA) cycle and passes electrons generated in this reaction along to the electron transport chain. In this review we will explore the recent evolution in our understanding of the underlying genetics and biology of SDH-deficient GISTs, and discuss treatment implications for these patients.

## Clinical Features of GISTs in the Pediatric Population

Gastrointestinal stromal tumor in children typically presents in the second decade of life (median age of 13 years) with a strong predilection toward females, who represent ∼70–75% of sporadic pediatric cases (Benesch et al., 2009; Pappo and Janeway, 2009; Rink and Godwin, 2009). This gender bias seems to be developmentally related: in a series of 44 pediatric and young adults (5–21 years) with GIST originating in the stomach, essentially all patients under 16 years of age were girls (24/25, 96%), while the post-pubertal gender imbalance was less pronounced (11/19 females, 58%) (Miettinen et al., 2005a). In non-biased sample sets the main site of presentation is gastric (∼75%), and these tumors are more often of epithelioid or mixed epithelioid/spindled cell morphology, in contrast to the adult form in which spindle-cell morphology predominates (Benesch et al., 2009). Pediatric GIST often presents with multi-focal nodular growth (Kerr et al., 1999; Prakash et al., 2005; Agaram et al., 2008a), which is rarely seen in adult sporadic cases. In addition, lymph node involvement is seen more often than in adult GIST, often in association with metastatic disease to liver and abdominal sites (Kerr et al., 1999; Agaram et al., 2008a; Miettinen et al., 2011). The natural history of these tumors nevertheless appears to be more indolent than adult GISTs, as patients can survive for many years with metastatic disease (Miettinen et al., 2005a). Interestingly, many of the characteristics of GISTs in the pediatric population (e.g., female predilection, predominance of gastric, multi-focal tumors of epithelioid morphology) are not as clearly defined in the more heterogeneous group of young adult (i.e., 21–30 years) GIST patients (Miettinen et al., 2005a; Prakash et al., 2005; Agaram et al., 2008a; Rink and Godwin, 2009), suggesting that some but not all of the patients in this age group may more properly be considered with the pediatric group.

The hallmarks of early onset multi-focal disease with a strong predilection for females are suggestive of a cancer pre-disposition syndrome. A summary of some of the characteristics of several described GIST syndromes is shown in Table 1. Familial GIST Syndrome (FGS), seen in ∼20 kindreds, is an autosomal dominant GIST tumor syndrome associated with germline *KIT* or *PDGFRA* mutations (Agarwal and Robson, 2009). FGS-associated GISTs resemble the sporadic kinase-mutant forms in terms of gender balance, anatomic distribution, and prevalence of spindle-cell morphology. GISTs occurring in the context of the autosomal dominant disorder neurofibromatosis type 1 (NF1, OMIM 162200), while generally WT for *KIT* and *PDGFRA*, typically present in the small bowel and possess spindle-cell morphology (Andersson et al., 2005; Miettinen et al., 2006a). GISTs in these patients also tend to manifest later in life, and these patients manifest the cutaneous and ocular findings associated with NF1 in addition to their pre-disposition for GISTs and various nervous system tumors. However, pediatric GISTs share a number of features with GISTs of the CT. CT, first described in 1977 (Carney et al., 1977) and subsequently reviewed in larger case series (Carney, 1983, 1999), is the non-familial association of gastric GISTs, functioning extra-adrenal paraganglioma (PGL), and pulmonary chondromas (OMIM 604287). Recently, esophageal leiomyomas and adrenal cortical adenomas have been added as components of the syndrome (Carney, 1999; Knop et al., 2006). Approximately 88% of CT patients are female with onset in the majority of patients before the age of thirty (Zhang et al., 2010). As with the pediatric patients with non-syndromic GIST, CT in males is generally seen in post-pubertal patients (Carney, 1983; Matyakhina et al., 2007). The GIST component of the triad predominately arises in the stomach and typically lacks detectable *KIT* and *PDGFRA* mutations. These and other shared characteristics (e.g., epithelioid morphology, chronic yet indolent clinical course) suggest a strong connection between sporadic pediatric GISTs and those associated with the triad. Indeed, the long interval (mean ∼8 years) (Carney, 2009) between detection of the first and second components of the CT imply that some apparently sporadic cases of pediatric GIST may actually be *formes frustes* of the triad.

**Table 1:** **Characteristics of syndromic GISTs**.

Syndrome	Carney triad	Carney–Stratakis	Familial GIST	NF1-related
Median age (years)	18	19	40–50	49
Gender predilection	Female > male	None	None	None
Germline mutations	Unknown	SDHB-D	KIT, PDGFRA	NF1
Inheritance	Not inherited	Autosomal dominant	Autosomal dominant	Autosomal dominant
Anatomic distribution	Gastric	Gastric	Gastric, small bowel	Small bowel
GIST histology	Epithelioid/mixed	Epithelioid/mixed	Spindle-cell	Spindle-cell

The coincidental occurrence of three uncommon tumors in young females suggested an etiological connection in the CT cases. The family histories of 79 patients with two or three of the associated tumors was examined (357 close relatives), only two cases with a familial connection were identified, indicating the triad is not inherited (Carney, 1999). Two patients with an incomplete presentation of gastric GISTs and PGL each had a sibling with multi-focal PGL. In a subsequent study Carney and Stratakis detailed a familial association of gastric stromal sarcomas (or GISTs) and PGLs in 12 patients (7 male and 5 female, average age 22 years) from five kindreds, including the original two sibships (Carney and Stratakis, 2002). In patients with the dyad of familial PGL and gastric GISTs, or CSS (OMIM 606864), the inheritance pattern appears to be autosomal dominant, albeit with incomplete penetrance. Familial PGL syndromes had long been documented (Chase, 1933; Chedid and Jao, 1974), however beginning in the 1990s linkage studies were able to map these syndromes to specific chromosomal loci (Heutink et al., 1992; Mariman et al., 1995; Niemann et al., 2001), and subsequently these syndromes were linked to germline mutations in component genes of the SDH complex (see Discussion below). In turn, germline *SDHB*, *SDHC*, or *SDHD* mutations were identified in the CSS families with the dyad of GIST and PGL, establishing the genetic basis of the disease (McWhinney et al., 2007; Pasini et al., 2008). These mutations have not been identified in the non-familial CT patients (Zhang et al., 2010). Recently, immunohistochemistry for the detection of an intact SDH complex has been used to triage genetic SDH deficiency in PGL, and utilized by groups working in the GIST field to solidify the involvement of the SDH complex in the oncogenesis of pediatric and syndromic GISTs, as well as a subset of adult WT GISTs. A flurry of recent reports has identified an important role for *SDHA* gene inactivation in SDH–deficient GIST.

## SDH Deficiency in GIST and in Associated Tumors

Both CT and CSS cases manifest the association of gastric GIST with extra-adrenal PGLs, so it is reasonable to suspect a shared etiology between these tumors in some cases. PGLs are highly vascularized tumors originating from neural crest-derived chromaffin cells. PGLs, along with pheochromocytomas (PHEOs) originating in the adrenal gland, are rare tumors, with an estimated annual incidence of 2–10 per million. As with GIST, most of these tumors occur sporadically, however up to 30% of these tumors may be inherited in a number of syndromes associated with specific gene mutations (Welander et al., 2011). PGLs and PHEOs, along with GISTs, are found in association with the autosomal dominant NF1 syndrome. However, as mentioned previously, NF1-asociated GISTs do not exhibit the clinico-pathological and demographic characteristics of CT and pediatric GISTs. Multiple endocrine neoplasia type 2 (MEN2, OMIM 171400), associated with PHEOs, medullary thyroid carcinoma, and other manifestations, is linked to gain-of-function mutations in the *RET* gene (Mulligan et al., 1993; Hofstra et al., 1994). *RET* encodes an RTK that plays an important role in neural crest development. Von Hippel–Lindau syndrome (OMIM 193300) is an autosomal dominant disorder that pre-disposes toward central nervous system and retinal hemangioblastomas, renal cell carcinomas, pancreatic tumors, along with PGL/PHEO and other manifestations. Germline and somatic bi-allelic mutations in the *VHL* gene serve to inactivate this tumor suppressor gene, which is involved in turnover of the hypoxia-inducible factors (HIF), part of the cellular transcription factor complex that responds to low oxygen (hypoxic) conditions (see Discussion below). To our knowledge *RET* and *VHL* mutations have not been reported in GIST, although several recent studies have looked for mutations in these genes in individual cases (Bano et al., 2012; Boguszewski et al., 2012). More recently, germline mutations in the transmembrane protein TMEM127 and in the transcription factor MAX (MYC associated factor X) have been linked to PHEOs and a few PGLs (Qin et al., 2010; Comino-Mendez et al., 2011; Welander et al., 2011). In the last decade, however, the involvement of the SDH complex in a number of hereditary PGL syndromes was clearly established, and in turn SDH deficiency was implicated in the oncogenesis of a subset of WT GIST.

Germline mutations in *SDHD* (Baysal et al., 2000), *SDHC* (Niemann and Muller, 2000), and *SDHB* (Astuti et al., 2001) are associated with the familial PGL/PHEO syndromes PGL1 (OMIM 16800), PGL2 (OMIM 601650), and PGL4 (OMIM 115310), respectively. More recently the PGL2 syndrome has been attributed to mutations in an assembly factor for the SDH complex, *SDHAF2* (Hao et al., 2009). In a recent development particularly relevant to the pathogenesis of SDH-deficient GISTs, a heterozygous germline *SDHA* mutation, accompanied by somatic loss-of-heterozygosity (LOH) in the 5p15 region surrounding *SDHA* was identified in a single PGL case (Burnichon et al., 2010). A second report identified mutations in this *SDH* gene in five additional PGLs among a group of 316 PGLs and PHEOs, including 129 apparently sporadic cases (Korpershoek et al., 2011). Deleterious mutations in any of these genes that result in loss of SDH catalytic activity have been shown to result in loss of expression of the SDHB protein (Douwes Dekker et al., 2003; Dahia et al., 2005). This phenomenon led to the development of a sensitive and specific approach to triage SDH deficiency based on negative immunohistochemical staining of the SDHB subunit (van Nederveen et al., 2009; Gill et al., 2010a). The utilization of SDHB IHC to identify SDH complex deficiency has recently been extended to the study of pediatric, syndromic, and adult WT GISTs, as summarized in Table 2. The initial study (Gill et al., 2010b) identified SDHB-deficiency in the gastric epithelioid GISTs from five CT cases and a single sporadic pediatric case, but not in the spindle-cell, non-gastric GISTs from seven young adults (age 21–29 years), or in three NF1-associated GISTs. Three SDHB-deficient tumors were identified in a panel of 103 consecutive sporadic adult GISTs. A second study (Gaal et al., 2011) confirmed SDHB negativity in six additional CT cases, as well as four cases with CSS. Five GISTs with *KIT* or *PDGFRA* mutations were all SDHB-positive, as were 41 of 42 apparently sporadic cases with unknown genotype. The sporadic adult SDHB-negative GISTs in both studies were gastric tumors of epithelioid morphology. In a larger study focused on GISTs with known *KIT*/*PDGFRA* genotype, SDH deficiency was investigated in 34 apparently sporadic WT GISTs from pediatric and adult patients, along with 18 *KIT* mutant GISTs and 5 NF1-associated GISTs (Janeway et al., 2011). Germline *SDHB-D* sequence analysis identified only three cases with pathogenic *SDHB* mutations and one case with an *SDHC* mutation. No mutations were found in the SDH assembly factor gene *SDHAF2* in 42 WT cases. Of the remaining 30 WT cases, all 18 pediatric GISTs were SDHB-negative, while 8/12 of the adult cases were SDHB-deficient. In contrast, 17/18 *KIT* mutant GISTs and 5 NF1-associated GISTs were positive for expression of the SDHB subunit. The authors were also able to demonstrate reduction of SDH complex enzymatic activity (succinate-cytochrome *c* reductase) in two SDHB-negative GISTs, comparable to that seen in an *SDHB*-mutant PGL. Notably, the adult WT sample set that was analyzed had a significant age bias (mean of 37 years, median 27 years): of the four cases with positive SDHB staining, three of those were over 55 years of age. In a subsequent study focused on determining SDH status in a large group of gastric GISTs, the authors arrived at a similar conclusion regarding the age-dependence of SDH-deficient GISTs (Miettinen et al., 2011). In a set of 756 gastric GISTs, 66 SDHB-negative GISTs were identified. When corrected for an age-specific selection bias in some of their cases, the estimated frequency of SDHB-negative gastric GISTs was 7.5%. Although SDHB-negative GISTs were found in patients up to 77 years of age, again these cases as a whole were much younger (mean 37 years, median 22 years) than the typical spectrum of GIST patients, and had an overall female predominance (71%). No mutations were detected in this large group of SDHB-negative GISTs, albeit only a limited panel of *SDHB*, *SDHC*, and *SDHD* exons was analyzed. The study also included 378 non-gastric GISTs, all of which were SDHB-positive. In another study of GISTs with known kinase genotype (Doyle et al., 2012), 42% of *KIT*/*PDGFRA* WT GISTs were negative for SDHB staining and were notably all gastric GISTs with multinodular architecture and epithelioid/mixed morphology. This result is interesting as it identifies a significant subset of SDH-positive adult WT GIST. In this study the large group of 170 *KIT*- and 32 *PDGFRA*-mutant GISTs were SDHB-positive. In summary, SDHB-deficiency is characteristic of all tested CT- and CSS-related GISTs, pediatric WT GISTs, and a minority of adult gastric GISTs, a population that is enriched when considering tumor characteristics such as multi-focal nodular architecture, an epithelioid cell component, and kinase WT status.

**Table 2:** **SDHB-deficiency in GISTs**.

Reference	Sample set analyzed	SDH-deficient^a^ (%)
Gill et al. (2010b)	5 CT	100
	1 Pediatric	100
	7 Young adult	0
	3 NF1-associated	0
	104 Sporadic	3
Gaal et al. (2011)	4 CSS	100
	6 CT	100
	5 Mutant	0
	42 Sporadic	2.3
Janeway et al. (2011)	2 *SDHX* mutants	100
	18 Wild type pediatric	100
	12 Wild type adult	67
	18 *KIT* mutant	6
	5 NF1-associated	0
Miettinen et al. (2011)	756 Gastric GISTs	8.7
	378 Non-gastric GISTs	0
Doyle et al. (2012)	179 KIT mutant	0
	32 PDGFRA-mutant	0
	53 Wild type	42

*^a^As determined by lack of SDHB protein immunoreactivity*.

## *SDHA* Mutations are Common in SDH-Deficient GIST

**Figure 1:**
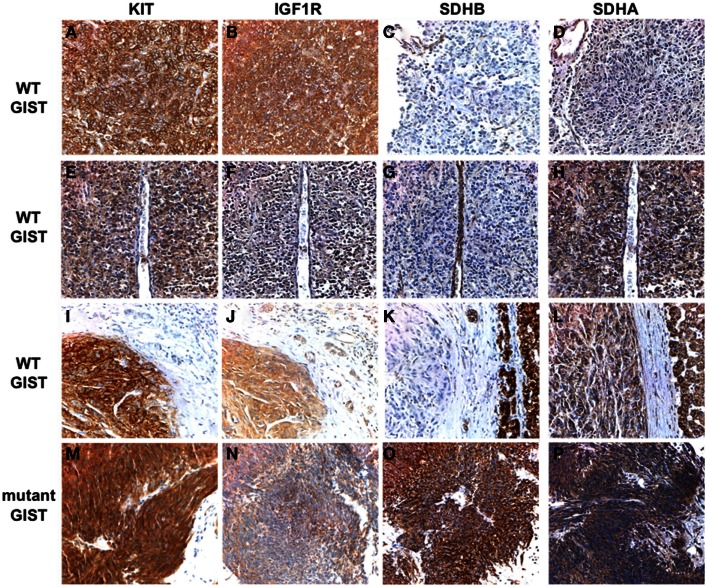
**Immunohistochemical analysis of KIT, IGF1R, SDHB, and SDHA expression in WT and *KIT* mutant GISTs**. Primary antibodies used include KIT (Dako), IGF1R (Cell Signaling), SDHB (Abcam), and SDHA (Abcam). Positive KIT staining is evident throughout tumor tissue in all cases **(A,E,I,M)**. Strong staining for IGF1R is seen in the WT GISTs **(B,F,J)** but not in the *KIT* mutant GIST **(N)**. SDHB staining is evident in the mutant GIST **(O)** and in the adjacent normal tissue and epithelial cells in the WT cases, but absent in the tumor tissue **(C,G,K)**. SDHA staining is absent in an SDHB-deficient GIST with a truncating *SDHA* mutation **(D)**. Positive SDHA staining is evident in an SDHB-deficient GIST harboring compound heterozygous missense *SDHA* mutations **(H)**, and in an SDHB-deficient GIST with no identified *SDH* mutations **(L)** as well as in the mutant GIST **(P)**. See text for more detailed mutation descriptions. GIST cases have been previously reported (Belinsky et al., 2012): **(A–D)**, case 2; **(E–H)**, case 1; **(I–L)**, case 10; **(M–P)**, case 21.

Succinate dehydrogenase B-deficiency in PGL has generally been connected to mutations in *SDHB*, *SDHC*, or *SDHD* (van Nederveen et al., 2009; Gill et al., 2010a), and, as previously noted, germline *SDHB-D* mutations were identified in most of the CSS GIST cases (Pasini et al., 2008). In the studies cited above, only a few *SDHB-D* mutations were described in sample sets totaling ∼140 cases of SDHB-negative GISTs. It should be noted that not all cases were subject to *SDHX* gene sequencing, some of the sequencing involved only a select panel of exons from *SDHB*, *SDHC*, and *SDHD*, and only a few cases in one study (Janeway et al., 2011) were examined for *SDHA* mutations. Sequencing *SDHA* is a technical challenge, due to its large size (664 amino acids encoded on 15 exons) as well as the presence of multiple pseudogenes on chromosome 3 (*SDHAP1*, *SDHAP2*) and chromosome 5 (*SDHAP3*). After the initial report of bi-allelic *SDHA* inactivation in a PGL case (Burnichon et al., 2010), two reports from the same group identified inactivating *SDHA* mutations in four unselected WT GIST cases, first by whole-transcriptome next-generation sequencing in two cases (Pantaleo et al., 2011a), and subsequently in two additional cases by conventional Sanger sequencing (Pantaleo et al., 2011c). The cases were WT non-gastric GISTs from three adults (age 26–38 years) and one pediatric patient, and in three of the four cases a germline nonsense or missense mutation was identified accompanied by inactivation of the second allele by LOH (one case) or a second heterozygous mutation (two cases). In the other case germline DNA was not available, however compound heterozygous missense mutations were identified in the GIST. Subsequently, in a sample set of 11 SDHB-negative WT GIST cases (all gastric GISTs with an epithelioid component), our group identified *SDHA* mutations in five cases (45%), as well as a single case with an *SDHC* mutation (Belinsky et al., 2012). The cases with *SDHA* mutations were adults (age 33–52 years) with apparently sporadic cases of GIST. Germline DNA was available in three cases, allowing us to demonstrate that *SDHA* inactivation was due to a germline mutation (nonsense or frameshift) plus an additional somatic point mutation (one case) or loss of the WT allele in the GIST (two cases). In the other two cases for which germline DNA was not available, sequencing of the tumor revealed homozygous missense *SDHA* mutations, presumably due to a germline point mutation accompanied by allelic losses surrounding the chromosome 5p15 locus, identified using high-density SNP copy-number arrays. Wagner et al. (2012) investigating a larger sample set of 33 SDHB-deficient GIST, used SDHA IHC to identify a subset of nine SDHA-deficient GISTs (27%). These patients included five men and four women with a median age of 38 years (range 19–53). The nine SDHA/SDHB-deficient GISTs all harbored inactivating *SDHA* mutations: in six cases DNA was available to demonstrate that one copy of the gene was inactivated in the patient’s germline. *SDHA* mutations have been reported in nine additional SDHA/B-deficient cases (Dwight et al., 2012; Italiano et al., 2012; Oudijk et al., 2012), adding to the perception of the utility of SDHA IHC in identifying cases with *SDHA* mutations. However, in a very recent study (Miettinen et al., 2012), three cases were reported in which tumors were SDHB-negative/SDHA-positive by IHC, yet harbored mutations in the *SDHA* gene. We have noted this phenomenon in one of our own cases, as shown in Figure 1. SDHB staining is absent in the tumor tissue of all three WT GISTs (Figures 1C,G,K), while the mutant GIST (Figure 1O) and normal cell compartments within the WT sections stain positive. The kinase WT GIST represented in the top panels has a homozygous, truncating *SDHA* mutation (c.457-3_c457delCAG, p.L153Kfs*71). This tissue as expected is immunonegative for SDHA (Figure 1D) as well as for SDHB. However, the second WT tumor, with compound heterozygous *SDHA* missense mutations (c.818C > T, p.T2731I; c1357G > A, p.G453R) stains positive for SDHA (Figure 1H), as does the third WT case, in which no *SDHX* mutations were detected (Figure 1L). The three cases reported by Miettinen et al. all possessed *SDHA* missense mutations, including the T273I mutation described in our case and two novel mutations (p.R188W, p.A454E). All these mutations fall within the FAD-binding domain of the SDHA flavoprotein and are predicted to be deleterious using *in silico* approaches based on sequence conservation and protein structure and function (Kumar et al., 2009; Adzhubei et al., 2010). These observations suggest that although SDHA-negative IHC is predictive of *SDHA* mutations, some missense mutations may escape detection using IHC alone.

**Figure 2:**
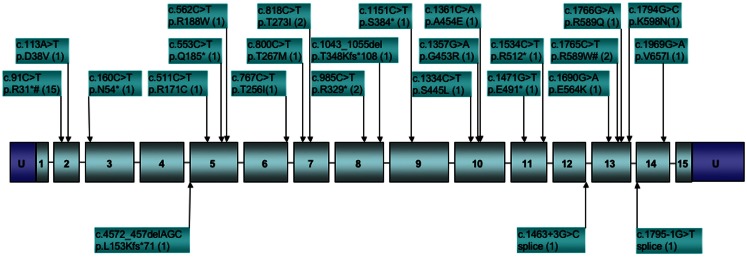
**Distribution of reported *SDHA* gene mutations in GIST**. Boxed numbers indicate exons, connecting lines represent introns (not drawn to scale), *U* = 5′, 3′ untranslated regions. Mutations are annotated at the cDNA and protein level, followed by the number of reported cases in parentheses. *Indicates stop codon. ^#^Mutations reported in paraganglioma.

To date, *SDHA* mutations have been reported in 37 GIST cases (Korpershoek et al., 2011; Pantaleo et al., 2011c; Belinsky et al., 2012; Dwight et al., 2012; Italiano et al., 2012; Miettinen et al., 2012; Wagner et al., 2012). In the five largest series reporting *SDHA* gene sequencing in SDHB-deficient GISTs (Belinsky et al., 2012; Dwight et al., 2012; Miettinen et al., 2012; Oudijk et al., 2012; Wagner et al., 2012), the frequency of reported *SDHA* mutations is ∼31%. While it is important to consider that the overall frequency of *SDHA* mutations in GIST is low given that SDH deficiency is seen in only ∼8% of all gastric GISTs (Miettinen et al., 2011), mutations in the *SDHA* subunit alone may be as common as mutations in all other subunits combined (Miettinen et al., 2012). This observation is perhaps not surprising given the relative size of the *SDHA* gene as compared to the other subunit genes, however this pattern is not seen in PGLs and PHEO, where *SDHA* mutations and allelic losses are rare as compared to those reported for *SDHB* and *SDHC* subunit genes (Burnichon et al., 2010; Korpershoek et al., 2011; Welander et al., 2011). This may point to a lack of penetrance of *SDHA* mutations in initiating PGL/PHEO neoplasms. For the reported *SDHA*-mutated GISTs, all originated in the stomach, generally with epithelioid/mixed morphology (one spindle-cell tumor was reported). Of the 27 cases for which individual patient data was reported, 17 (63%) were female, with a median age of 38 years (range 14–53). This agrees well with the summary reporting by Miettinen et al. (2012) for 36 SDHA-deficient cases, in which 64% of cases were female with a median age of presentation of 34 years. Altogether 25 distinct *SDHA* mutations have been identified in GIST cases, as summarized in Figure 2. As can be seen from the figure, missense, nonsense, and splice-site mutations, along with small deletions, have been identified within or adjacent to the coding region of 12 of the 15 exons of the gene. The one notable hotspot is the R31* mutation in exon 2, representing 15/42 or ∼36% of the occurrences. In most cases bi-allelic inactivation was detected, generally due to an LOH event, although in several cases compound heterozygous *SDHA* mutations were reported (Pantaleo et al., 2011c; Belinsky et al., 2012; Dwight et al., 2012; Wagner et al., 2012). Notably, in a few cases where a heterozygous *SDHA* mutation was reported, a second genetic event was not detected (Dwight et al., 2012; Italiano et al., 2012; Oudijk et al., 2012; Wagner et al., 2012), leaving open the possibility that only one allele was inactivated, or that some other mechanism, genetic or epigenetic, was responsible for bi-allelic inactivation. Along these lines, in our own study we reported one case with a heterozygous somatic mutation in the *SDHC* gene (p.Gly75Asp) that was fully homozygous in cDNA (Belinsky et al., 2012). In this one case, we were able to provide convincing evidence for silencing of the remaining WT allele through hyper-methylation of a CpG island present at the 5′ end of the gene (Huang et al., 2009).

## Distinct Patterns of Cytogenetic Progression in SDH-Deficient GIST

Although GISTs are generally considered cytogenetically non-complex (Yang et al., 2008), the oncogenetic progression of sporadic GISTs with activating kinase mutations is usually accompanied by characteristic, large-scale chromosomal copy-number aberrations (CNAs). In primary GISTs the mean number of CNAs detected is ∼7 per tumor, with losses outnumbering gains by ∼2.5-fold (Wozniak et al., 2007). Loss of all or part of chromosome 14q is a frequent (∼70%) and apparently early event in GIST, seen in both benign and malignant tumors (el-Rifai et al., 1996; Breiner et al., 2000; Debiec-Rychter et al., 2001; Gunawan et al., 2007). Early studies, combining cytogenetic, chromosomal comparative genomic hybridization (CGH), and microsatellite LOH approaches, delineated specific regions of CNA on 14q, including 14q11–q12, 14q22–q23, and 14q24.3 (El-Rifai et al., 2000b; Debiec-Rychter et al., 2001). Copy-number losses on chromosome 22q and 1p are also common, seen in about 50% of GISTs (El-Rifai et al., 2000a; Chen et al., 2004; Gunawan et al., 2007). Chromosome 22q deletions were targeted in additional studies to identify common deleted regions at 22q13.3 and 22q11.22 (Lasota et al., 2005) and at 22q12.2 and 22q12.1 (Pylkkanen et al., 2003). These CNAs on chromosomes 1, 14, and 22 seem to occur in tumors with either *KIT* or *PDGFRA* mutations (Heinrich et al., 2003; Assamaki et al., 2007; Wozniak et al., 2007), but exhibit distinctive tumor site-dependence, with 14q losses more common in gastric GIST, while 1p (and 15q) losses are seen more often in intestinal GISTs (Gunawan et al., 2004; Wozniak et al., 2007). The pattern of aberrations in a large group of 116 gastric and 87 intestinal GISTs was used to generate an “oncogenic tree model” describing the cytogenetic evolution of these GISTs. This model identified three pathways of cytogenetic evolution (e.g., pathways initiated by 14q, 1p, or 22q loss), with distinctive characteristics regarding tumor-site, genomic complexity, and prognosis (Gunawan et al., 2007). In addition to these common changes, copy-number losses have also been described with significant frequency on chromosomes 9, 13q, 10q, and 21q (El-Rifai et al., 2000a; Chen et al., 2004; Wozniak et al., 2007; Belinsky et al., 2009). Some of these chromosomal losses (e.g., 9p and 9q, 13q, and 15q) are more often seen in advanced or metastatic GIST and may have value as prognostic markers (El-Rifai et al., 2000a; Kim et al., 2000; Gunawan et al., 2004; Wozniak et al., 2007). Similarly, regions of chromosomal copy-number gain, specifically gains in chromosome 5 and 8, while they occur less frequently overall in GIST, seem to be characteristic of more advanced GISTs (El-Rifai et al., 2000a; Debiec-Rychter et al., 2001; Assamaki et al., 2007). Array-CGH studies of fairly large sample sets of *KIT* and *PDGFRA*-mutated tumors have refined the minimum overlapping regions commonly deleted in GISTs, including 1p, 14q, 15q, and 22q, along with some less common regions of copy-number loss and gain (Assamaki et al., 2007; Wozniak et al., 2007). More recently, the combination of high-resolution genome-wide copy-number analyses with global gene expression data has been used to develop integrated molecular portraits of the oncogenesis of *KIT*/*PDGFRA*-mutated GIST (Astolfi et al., 2010; Yang et al., 2010; Ylipaa et al., 2011).

The SDH-deficient gastric GISTs found in children, either sporadically or in the context of CT, appear to lack the characteristic chromosomal losses of mutant GIST, and generally are less cytogenetically complex than the mutant GISTs that comprise the majority of adult sporadic cases. In an early report analyzing GISTs from three pediatric/young adult female CT cases, CGH analysis identified one region of chromosome loss (13q32–q34) in one of the three GISTs, along with 13 regions of CN gain/amplification in the three tumors (Agaimy et al., 2007). In a larger CGH study of 31 GISTs (plus 10 associated tumors, mainly PGLs) from 28 CT patients (including 13 pediatric cases), fully 55% of the GISTs showed no significant CGH changes (Table 3) (Matyakhina et al., 2007). Losses at 14q or 22q were identified in only one GIST case each. The 31 GISTs analyzed manifested a mean of only ∼1.1 CNAs per tumor (range 0–6). Interestingly, recurrent losses were identified on chromosome 1q (four cases) or 1p (four cases), or both (two cases). While the *SDHB* gene (1p36.1–p35) and *SDHC* gene (1q23.3) reside on chromosome 1, involvement of these genes in the reported CT cases is unclear, as no mutations were identified in *SDHA-D* genes in this study. In a study of 13 pediatric GIST cases, including two CT patients, low-density SNP arrays were used to identify chromosomal CNAs and regions of LOH (Table 3) (Janeway et al., 2007). These tumors showed even less cytogenetic progression, with 10/13 (77%) showing no detectable changes. Only seven regions of CNA or LOH were reported in the 13 cases, despite the fact that the sample set included high risk or recurrent/metastatic pediatric GIST. In the case of adult WT GIST, a few cases have been reported with significant cytogenetic progression (Assamaki et al., 2007; Belinsky et al., 2009). However, in our recent study of 16 *SDH*-deficient GIST from 11 cases (including 3 CT cases), most of which were sporadic adult cases, a total of 30 CNAs or LOH events were identified (∼1.8/samples). No 14q/22q losses were seen at all, and only one case exhibited chromosome 1p loss. Recurrent losses or LOH events at chromosome 5p (five cases) were generally found in the cases with *SDHA* coding-region mutations (Table 3). Similarly, allelic LOH events were documented in association with germline *SDHB-D* mutations in CSS patients (McWhinney et al., 2007; Pasini and Stratakis, 2009), and more recently in additional *SDHA*-mutated GISTs (Pantaleo et al., 2011c; Dwight et al., 2012; Oudijk et al., 2012; Wagner et al., 2012). As mentioned previously, the SDHB-deficient GISTs seen in young sporadic and CT cases generally exhibit an indolent yet unpredictable clinical course (Miettinen et al., 2005a; Agaimy et al., 2007; Agaram et al., 2008a; Zhang et al., 2010). It is tempting to speculate that the lack of cytogenetic progression exhibited by SDH-deficient GISTs may in part account for their more indolent clinical behavior in comparison to kinase-mutant GIST. To this point it is interesting to note that in the large CGH study of CT patients, metastatic lesions showed a trend toward greater cytogenetic progression than benign tumors (Stratakis and Carney, 2009). To reach a better understanding of the clinical behavior of WT GIST may require further investigation of the molecular genetic, cytogenetic, and epigenetic changes in these tumors. To this point, a very recent report describes the epigenomic divergence between SDH-deficient WT GIST and GISTs with activating kinase mutations (Killian et al., 2013). In this study, a set of 24 SDH-deficient GISTs, again with relatively stable genomes, was found to exhibit a pattern of global DNA hyper-methylation, in comparison to 39 RTK mutant GISTs. This pattern of epigenomic divergence was also seen in SDH-deficient PGL/PHEO, as well as in gliomas exhibiting defects in another Krebs cycle enzyme, isocitrate dehydrogenase (IDH), establishing a link between Krebs cycle metabolism and maintenance of methylation patterns in genomic DNA.

**Table 3:** **Reported chromosomal aberrations in wild type GIST**.

Reference	Sample set analyzed	Methodology	Notable findings
Matyakhina et al. (2007)	31 GISTs from 28 CT cases^a^	CGH	No changes reported in 17 GISTs (55%)
			Frequent 1p and/or 1q losses (six cases each, 19%)
			Additional changes reported in ≤2 cases:
			Gains: 1p11, 1q, 4p11–p15, 5p11–q11, 8q33–q34, 12p11–p12, 12q13–q22, 15q14-qter
			Losses: 3q13–qter, 6q15–qter, 10q22–qter, 11p, 14q23–qter, 16q, 17p, 17q, 22q
Janeway et al. (2007)	13 Pediatric GISTs^b^	SNP array	No changes reported in 10 GISTs (77%)
			One case with 5p gain only
			One case with copy-number neutral 11q LOH only
			One case with multiple regions of LOH (1p, 3q, 5q, 13, 18)
Belinsky et al. (2012)	16 SDH-negative GISTs from 11 cases^c^	SNP array	No changes reported in two cases (18%)
			5p Losses/LOH in five cases (45%)
			1q Gains in three cases (27%)
			Additional changes reported in ≤2 cases:
			Gains: 5p15.33.q35.3, 11p15.5.p15.3, 11q12.3.q22.3
			Losses: 1p36.33.p12, 10p15.3.126.3, 11q22.3.q25, 13q11.q34, 17p12, 19p12, 22q11.23

*^a^Includes 13 pediatric cases*.

*^b^Includes two CT cases*.

*^c^Includes three CT cases (one pediatric, two adult)*.

## Expression of IGF1R in SDH-Deficient WT GISTs

As mentioned above, the great majority of pediatric GISTs lack mutations in either *KIT* or *PDGFRA*, and a significant subset of adult GIST is also WT for these RTKs. However, most pediatric and WT GISTs express KIT (Figures 1A,E,I), and several studies suggest that the KIT receptor and downstream signaling pathways are activated in pediatric GISTs (Janeway et al., 2007; Agaram et al., 2008a). Two early studies from the same group endeavored to characterize pediatric GISTs using global gene expression profiling (Prakash et al., 2005; Agaram et al., 2008a). In the initial study, the gene expression patterns of seven gastric GISTs from two pediatric WT cases and two young adults (one WT, one mutant) were compared to 10 gastric GISTs from adults (one WT, nine mutant). Unsupervised clustering approaches identified distinct transcriptional profiles for the GISTs from the pediatric/young adult cases, which with one exception clustered separately from the adult cases. This analysis identified 385 differentially expressed genes between the two groups (Prakash et al., 2005). In the second study, a larger group of 13 gastric tumor nodules, all WT, from 8 pediatric WT cases was analyzed (several of these cases were analyzed in the first report). The pediatric group included two CT cases. Two comparisons were made, first to 5 WT GISTs from adult patients, then to a set of 19 primary GISTs from adults of mixed genotype (3 WT, 4 *PDGFRA*-mutants, 12 *KIT* mutants), but matched as to gastric location. In both analyses the pediatric and adult cases formed distinct clusters based on their transcriptional profiles, with the notable exception of three young adult cases (<30 years) with clinico-pathological characteristics more in keeping with the pediatric GISTs (e.g., multi-focality, epithelioid cell morphology, indolent clinical course). These three tumors (two WT, one *KIT* exon 11 mutant) clustered instead with the pediatric WT tumors. An intersection of genes in common to the two analyses identified 814 differentially expressed genes in the pediatric group (Agaram et al., 2008a). Intriguingly, of the genes reported in the two studies, the only gene in common was another RTK, the insulin-like growth factor receptor (IGF1R), which was expressed 11–14 fold higher in the pediatric GIST samples. In 2008 we reported that IGF1R was over-expressed at the protein and RNA level in several adult WT GIST samples, in comparison to mutant GISTs (Tarn et al., 2008). We subsequently confirmed this observation in a larger set of mainly adult WT GISTs (Belinsky et al., 2008), while Janeway et al. (2010) reported the over-expression of IGF1R protein in pediatric WT GISTs. In these studies, the levels of total and phosphorylated IGF1R protein in WT GISTs do not correspond well, perhaps due to cross-reactivity of the phospho-IGF1R antibody with the phosphorylated insulin receptor (Janeway et al., 2010). The over-expression of IGF1R RNA is not usually accompanied by amplification of the gene (Pantaleo et al., 2009; Janeway et al., 2010; Chou et al., 2012). Instead, the high level of IGF1R expression in pediatric GISTs and a subset of the WT GISTs found in adults may be part of a transcriptional profile inherent to the cell of origin of these distinctive GISTs. Pantaleo et al. (2011b) recently reported an expression profiling study of four gastric WT GISTs from young adult cases, along with 26 mutant GISTs, and a meta-analysis of these expression profiles versus published data for murine ICCs. Interestingly, the expression data from human mutant GISTs and murine ICC clustered together, and separate from the WT tumors. An analysis of the top 448 differentially expressed genes (with *P* < 0.001) with respect to gene ontology and tissue expression revealed a pattern of expression within the WT tumors of genes related to neurogenesis functions and derived from neural tissues. IGF1R was again among these genes, and the over-expression of IGF1R, as well as that of several other neural markers, was validated in the study. This data adds support to the proposal that WT GISTs may derive from IGF1R-expressing ICC progenitor cells, rather than mature ICCs (Lorincz et al., 2008).

With the increased emphasis on the role of SDH deficiency and *SDHX* mutations in pediatric, syndromic, and adult WT GISTs, several recent studies have looked at the correlation between IGF1R expression and SDH deficiency. In our own study, 11 of 12 WT GIST samples were SDHB-negative: all these tumors over-expressed IGF1R at both the RNA and protein level (see for illustration Figures 1B,F,J as compared to Figure 1N). The one SDHB-positive WT tumor expressed IGF1R at comparable levels to the mutant GISTs in the study (Belinsky et al., 2012). Chou et al. (2012) recently reported a study of eight SDH-deficient GISTs along with 3 NF1-related and 40 unselected tumors (35 mutant, 5 WT). Two of the unselected WT GISTs were also SDHB-negative; these 10 SDHB-negative tumors all expressed high levels of IGF1R as evaluated by IHC. In contrast, the remaining WT GISTs, along with the 3 NF1-derived and 35 mutant tumors, expressed the receptor at low levels. The strong correlation of IGF1R expression and SDH status has been confirmed with a large IHC study of 1078 GIST, including 705 gastric GISTs and 373 originating in the intestine (Lasota et al., 2012). Eighty SDHB-negative GISTs, all originating in the stomach, were identified: of these, 71 were high IGF1R-expressing tumors. Of 625 SDH-positive gastric GISTs, only 9 expressed significant levels of IGF1R. All intestinal GISTs were SDHB-positive and expressed low IGF1R levels. From these studies it seems clear that IGF1R over-expression is a further defining characteristic of the SDH-deficient WT gastric tumors found mainly in children and in younger adults.

## Therapeutic Opportunities for WT SDH-Deficient Pediatric and Adult GISTs

Wild type SDH-deficient GISTs constitute the great majority of pediatric cases and a significant percentage of gastric occurrences in younger adults. It is reasonable to expect that the targeted therapies that have proven effective in mutant GISTs may prove inferior as systemic therapy in tumors lacking gain-of-function kinase drivers. Historical data in patients with advanced GIST suggests that median overall survival (OS) without TKI therapy is 20 months and that cytotoxic chemotherapy is not effective (Gold et al., 2007). Standard approved therapies for GIST now consist of the TKIs IM and sunitinib. Therapy with IM provides response rates of ∼72% and improved median OS of >60 months in adult patients with GISTs harboring *KIT* exon 11 mutations, while WT patients exhibited response rates of only ∼45%, and OS less than 50 months (Heinrich et al., 2008b). In adults with metastatic GIST that had progressed on IM, clinical benefit of sunitinib was superior for patients with WT versus *KIT* exon 11 mutations (56 versus 34%), however no objective responses were documented (Heinrich et al., 2008a). For children with GIST, of which the great majority are WT tumors, the primary treatment remains surgical resection of the tumor with testing and removal of involved lymph nodes, followed by postoperative surveillance using CT or MRI scans (Janeway and Pappo, 2012). There have been recent reports evaluating the use of IM and sunitinib in pediatric GIST patients (Cypriano et al., 2004; Hayashi et al., 2005; Kuroiwa et al., 2005; Prakash et al., 2005; Sauseng et al., 2007; Agaram et al., 2008a; Bond et al., 2008; Delemarre et al., 2008; Janeway et al., 2009). These studies revealed only one documented response to IM in a 14-year-old male with CT (Delemarre et al., 2008) and additional instances of stable disease for periods of 6–16 months. Sunitinib appears to be slightly more effective. Partial response (PR) has been reported in two young patients (ages 10 and 18 years) with WT GIST but the more typical response to sunitinib in pediatric patients with WT GIST is SD (Agaram et al., 2008a; Janeway et al., 2009). However, time to progression on sunitinib was longer than time to progression for previous IM in most of these patients (Janeway et al., 2009).

As described in several recent reviews, other TKIs are being tested in clinical trials for efficacy as systemic therapy in GIST patients who progress or show intolerance to IM/sunitinib (Demetri, 2011; Kim and Zalupski, 2011). These agents differ in properties such as mode of binding, kinase selectivity, and other biological properties, some of which may prove advantageous in the treatment of WT pediatric and adult GISTs. For example, masitinib (AB Science), an oral TKI with selectivity for KIT and PDGFRA, inhibits WT KIT at sub-micromolar concentrations in cell-based assays (Dubreuil et al., 2009). In a Phase II trial testing masitinib as front-line therapy for 30 patients, including 3 WT cases, masitinib treatment resulted in CR/PR in 16 patients and stable disease in 13 others; the mutation-dependent response has not yet been reported (Le Cesne et al., 2010). Other RTKs exhibiting greater *in vitro* activity against WT KIT than IM, including dasatinib (Sprycel, Bristol–Meyers Squibb), sorafenib (Nexavar, Bayer), and nilotinib (Tasigna, Novartis) (Agaram et al., 2008a) have been investigated, but again much of the data on benefit has not been correlated with mutational status.

With the significant progress made during the last few years in elucidating the unique biology of WT GISTs (both pediatric and adult), novel targets have come to light, which may have potential therapeutic implications. First, as detailed above, IGF1R is over-expressed in the vast majority of SDH-deficient WT GISTs, including pediatric cases, although some WT GISTs express the receptor at levels comparable to *KIT*/*PDGRA* mutant tumors (Corless et al., 2009). With respect to IGF1R and its downstream signaling molecules, our group has showed that the small molecule TKI, NVP-AEW541 (Novartis), which has activity against IGF1R, can lead to cytotoxicity in mutant GIST cell lines, via AKT and MAPK signaling that is independent from KIT signaling. Similar findings were observed when IGF1R levels were impaired using targeted siRNAs. Additive effects were observed by combining NVP-AEW541 and imatinib, suggesting a potential therapeutic benefit in targeting IGF1R in GISTs that are unresponsive to imatinib, including GISTs that overexpress IGF1R (Tarn et al., 2008). It should be noted however that these studies were performed in *KIT* mutant cell lines. Fortunately, a number of agents targeting IGF1R were already being developed and tested in solid tumors, allowing for rapid translation from the bench findings to ongoing clinical trials in GIST. A recent report published the first anti-IGF1R-targeted therapy in a patient with NF1-associated WT GIST (Day et al., 2011). This patient progressed within 4 weeks of treatment with the anti-IGF1R fully humanized monoclonal antibody R1507 (Roche). NF1-associated WT GISTs, as noted above, are generally positive for SDHB expression and express low levels of IGF1R (Gill et al., 2010b; Janeway et al., 2011; Chou et al., 2012). In the reported case, it was determined that the tumor had very low expression of IGF1R, a plausible explanation for the lack of efficacy in this patient. An ongoing phase II trial is testing the IGF1R inhibitor linsitinib (OSI-906, Astellas, NCT01560260) in GIST patients that lack mutations in *KIT*, *PDGFRA*, and *BRAF*.

**Figure 3:**
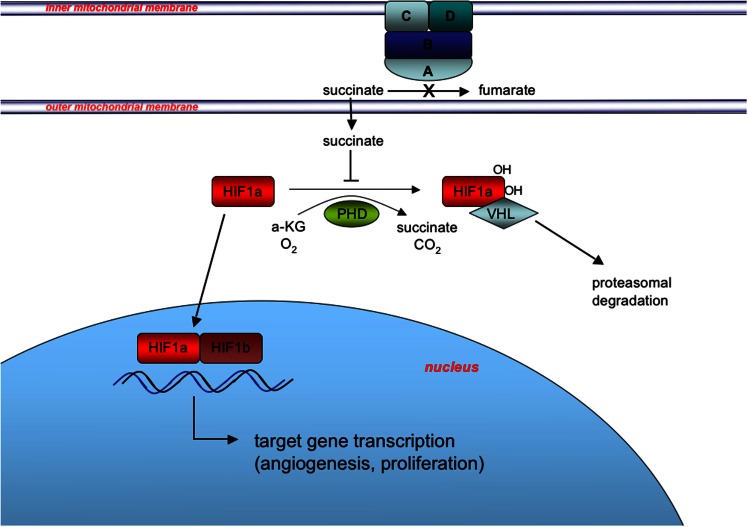
**Induction of the pseudohypoxic response in SDH-deficient GIST**. SDH, composed of the catalytic subunit (SDHA and SDHB) anchored to the inner mitochondrial membrane through subunits SDHC and SDHD, oxidizes succinate to fumarate as part of the tricarboxylic acid (TCA) cycle and couples this oxidation to the reduction of Coenzyme Q (CoQ, ubiquinone) as complex II of the electron transport chain (not shown). Inactivation of SDH via *SDHX* gene mutation or other mechanisms leads to succinate accumulation in the mitochondria and subsequent export to the cytosol via metabolite transporters (not shown). Increased cytosolic succinate levels act to stabilize one of three HIFα subunits (HIF1α is shown for simplicity) via product inhibition of PHD-mediated hydroxylation of HIF1α, which skirts the VHL-mediated targeting of HIF1α for degradation. Stabilization and nuclear translocation of HIF1α leads to formation of the heterodimeric HIF transcription factor with constitutively expressed nuclear-located HIF1β, and induction of the pseudohypoxic response.

The more recent findings that many WT GISTs have deficiencies in SDH suggest the possibility of exploiting a second target pathway, namely the hypoxic response to SDH complex inactivation that is facilitated by HIF transcriptional factors. The SDH, anchored in the mitochondrial inner membrane, converts succinate to fumarate as part of the TCA cycle, and couples this oxidation reaction to the reduction of Coenzyme Q (CoQ, ubiquinone) (Figure 3). When SDH is inactivated by *SDH* mutation or other mechanisms, succinate accumulates in the mitochondria and is transported to the cytosol, where it has been shown to inhibit the enzyme prolyl 4-hydroxylase (PHD) (Selak et al., 2005). PHD belongs to a super-family of enzymes that couple hydroxylation of their substrate with oxidative decarboxylation of alpha-ketoglutarate to succinate (Schofield and Zhang, 1999; Ozer and Bruick, 2007). In normoxic conditions, PHD catalyzes the hydroxylation of HIFα, which facilitates binding to the *VHL* (von Hippel–Lindau) gene product and targeting of the HIFα for ubiquitination and proteasomal degradation. Under hypoxic conditions PHD activity is limited by oxygen deprivation allowing HIFα to accumulate (Pugh and Ratcliffe, 2003), while in pseudohypoxia induced by SDH deficiency, increased cytosolic levels of succinate lead to product inhibition of PHD (Selak et al., 2005). Stabilized HIFα translocates to the nucleus where it dimerizes with HIFβ subunits, and activates transcriptional programs promoting angiogenesis, glycolysis, and cell proliferation (Cervera et al., 2008).

Succinate dehydrogenase mutations leading to the activation of a pseudohypoxic response, and in particular up-regulation of the vascular endothelial growth factor (VEGF) have been described in other tumors (PGL and renal cell carcinoma) (Ricketts et al., 2008; Pasini and Stratakis, 2009; Favier et al., 2012; Linehan and Ricketts, 2012). Consistent with this mechanism, WT GISTs have been shown to have higher VEGF expression than their KIT/PDGFR-mutant counterparts (Antonescu et al., 2004). This suggests that targeting this pathway may be of interest and is supported by the limited data on sunitinib, which is a potent inhibitor of VEGFR (Demetri, 2011). In addition, a phase I study of nilotinib, which has activity against BCR-ABL, discoidin domain receptor (DDR), KIT, PDGFR, and colony-stimulating factor receptor-1 (CSF-1R) tyrosine kinases (Blay and von Mehren, 2011), showed remarkable disease stabilization, following failure of IM and sunitinib, in two younger adult women (ages 27, 39 years) whose GISTs harbored *SDHA* mutations (Pantaleo et al., 2012). These findings are intriguing since nilotinib had been shown to perform no better than imatinib in the majority of GIST patients enrolled on trial (CAMN107A2201-ENESTG3 and CAMN107G2301-ENESTG1) (Cauchi et al., 2012). These authors speculate that potential off-target effects of nilotinib could be targeting the pseudohypoxic response caused by the *SDHA* mutation. More studies examining the role of nilotinib on the treatment of SDH-deficient GISTs are warranted. Other RTKs with dual activity against KIT and the VEGF receptors include sorafenib, vatalanib, motesanib, and regorafenib, which was recently FDA approved for treatment of GISTs refractory to standard therapy in 2013 (Kim and Zalupski, 2011). The monoclonal antibody bevacizumab is another agent that targets angiogenesis, specifically by binding circulating VEGF. Finally, other agents that specifically target HIF1α level and/or signaling have been reported in various pre-clinical and clinical stages (Tennant et al., 2010).

## Conclusion

Gastrointestinal stromal tumor, once known as a chemotherapy refractory sarcoma, has become the poster child for targeted therapy. While the progress made in the clinic with TKIs has been profound, patients whose GISTs are not well controlled have become the focus of ongoing investigations. The rare cases of GISTs in the pediatric population were a conundrum, as they did not have the anticipated response to IM. We also noted 10–15% of patients in the adult population with metastatic disease who progressed rapidly within 6 months of IM therapy, some of whom had WT GISTs. Studies of these refractory pediatric patients have found parallels in the group of adult patients whose tumors do not have activating kinase mutations. It is now clear that differentiating these two groups based on the age of diagnosis is unwarranted. Research over the past decade, reviewed above, has led to the reclassification of some of these WT GISTs as SDH-deficient GISTs, whose hallmarks include gastric site of origin that is often multi-focal with lymph node involvement and with epithelioid histology. Future trials of GIST patients will need to consider these patients as biologically distinct and requiring strategies not focused on KIT and PDGFRA. In addition, this group of patients is now also one in whom genetic counseling must be considered given the risk of the familial CSS and its associated risk for malignant PGLs.

## Conflict of Interest Statement

The authors declare that the research was conducted in the absence of any commercial or financial relationships that could be construed as a potential conflict of interest.
